# Supplementary material for: Comparing Oncological and Perioperative Outcomes of Open versus Laparoscopic versus Robotic Radical Nephroureterectomy for the Treatment of Upper Tract Urothelial Carcinoma: A Multicenter, Multinational, Propensity Score-Matched Analysis
Source: Cancers (Basel). 2023 Feb 23;15(5):1409. doi: 10.3390/cancers15051409 (PMC10000228; doi:10.3390/cancers15051409)
Supplement: Supplementary file 1 [file cancers-15-01409-s001.zip › cancers-2164551-supplementary.pdf]

cancers MDPI

Supplementary material: Comparing oncological and perioperative outcomes of open versus laparoscopic versus robotic radical nephroureterectomy for the treatment of upper tract urothelial carcinoma: A multicenter, multinational, propensity score-matched analysis

Nico C. Grossmann, Francesco Soria, Tristan Juvet, Aaron Potretzke, Hooman Djaladat, Alireza Ghoreifi, Eiji Kikuchi, Andrea Mari, Zineddine Khene, Kazutoshi Fujita, Jay D Raman, Alberto Breda, Matteo Fontana, John P. Sfakianos, John L. Pfail, Ekaterina Laukhtina, Pawel Rajwa, Maximillian Pallauf, Cédric Poyet, Giovanni E. Cacciamani, Thomas van Doeveren, Joost L. Boormans, Alessandro Antonelli, Marcus Jamil, Firas Abdollah, Guillaume Ploussard, Axel Heidenreich, Enno Storz, Siamak Daneshmand, Stephen A. Boorjian, Morgan Rouprêt, Michael Rink, Shahrokh F. Shariat and Benjamin Pradere

Table S1. Overview of the number of missing and imputed variables of the initial dataset.

Variable	Observations per m			
	Complete	Incomplete/missing	Imputed	Total
Age	2430	4 (0.2%)	4	2434
Gender	2431	3 (0.1%)	3	2434
ASA	1895	539 (22.2%)	539	2434
BMI	1849	585 (24%)	585	2434
Previous bladder cancer	2201	233 (9.6%)	233	2434
Hydronephrosis	1972	462 (19%)	462	2434
Diagnostic ureteroscopy performed	2043	391 (16.1%)	391	2434
Tumor location	2109	325 (13.4%)	325	2434
Year of surgery	2256	178 (7.3%)	178	2434
Side	2230	204 (8.4%)	204	2434
Lymphadenectomy performed	2280	154 (6.3%)	154	2434
Blood loss	1539	895 (36.8%)	895	2434
Perioperative intravesical chemotherapy instillation	1672	762 (31.3%)	762	2434
Surgery duration	1202	1232 (50.6%)	1232	2434
Pathological tumor grade	2355	79 (3.2%)	79	2434
Pathological multifocality	2352	82 (3.4%)	82	2434
Number of lymph nodes removed	2264	170 (7%)	170	2434
Number of positive lymph nodes	2265	169 (6.9%)	169	2434
Soft tissue surgical margins	1900	534 (21.9%)	534	2434
Variant histology	2167	267 (11%)	267	2434

Number of patients with major complications	1894	540 (22.2%)	540	2434
Highest complication	1836	598 (24.6%)	598	2434
Length inpatient stay	1645	789 (32.4%)	789	2434
Adjuvant chemotherapy	2431	3 (0.1%)	3	2434
Adjuvant RT	1846	588 (24.2%)	588	2434
Bladder recurrence	2276	150 (6.2%)	150	2434
Bladder recurrence free survival	2276	150 (6.2%)	150	2434

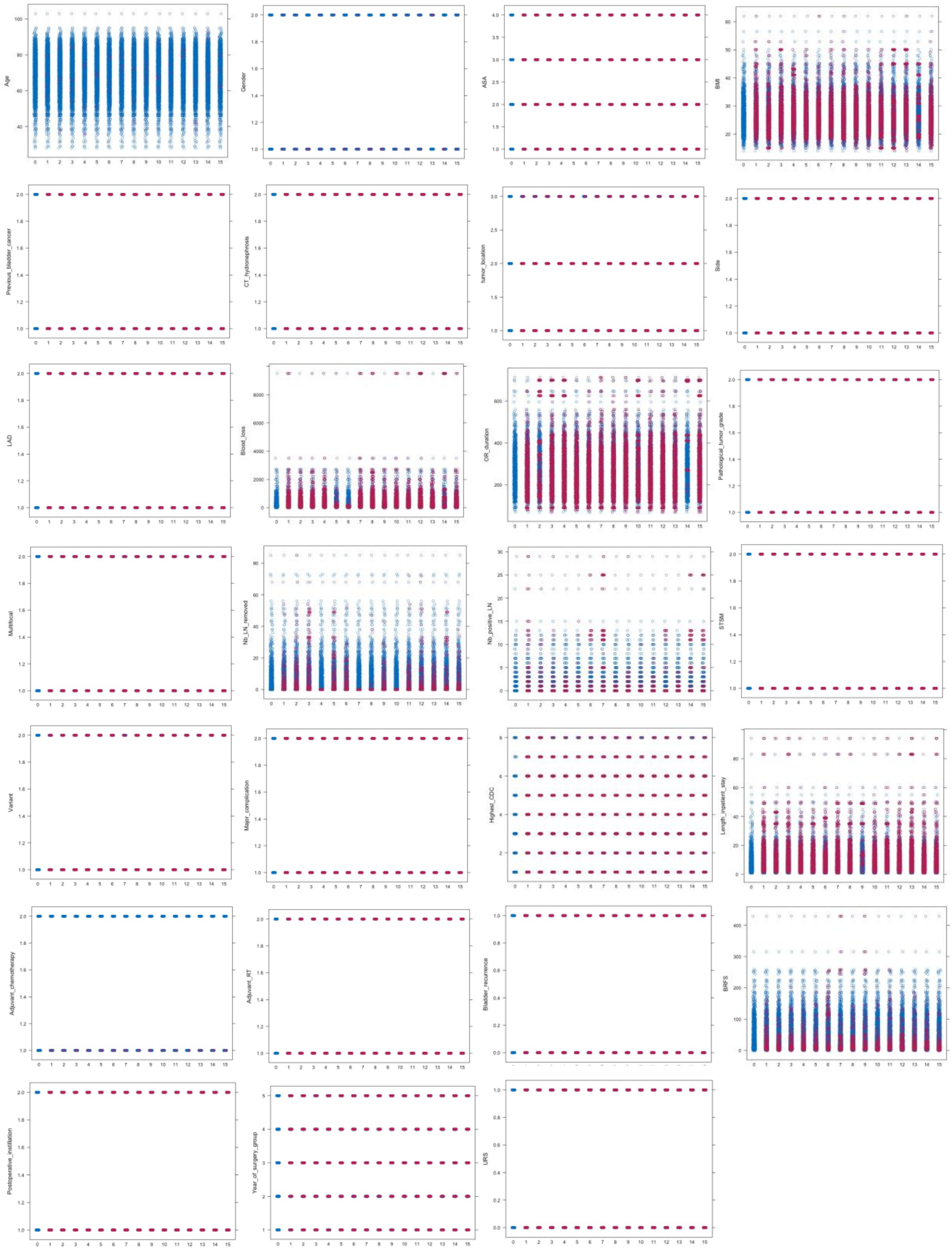

Figure S1: Strip plots depicting the discrepancy between the observed and imputed data. Specifying a maximum number of iterations of 50 and the number of imputation datasets of 15. The blue dots represent the original data and the red dots the imputed data.

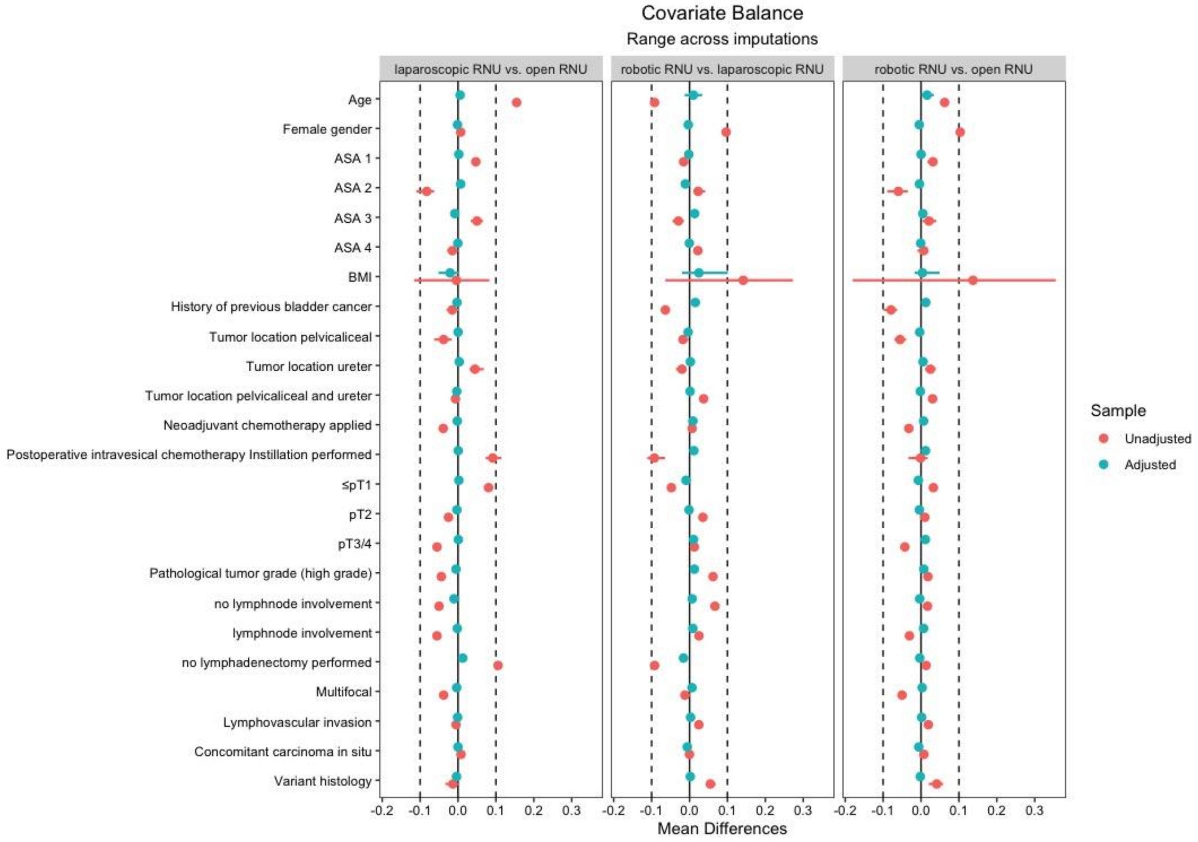

Figure S2. Love plot indicating the balance before and after propensity score matching across all 15 imputations.
